# Cutaneous Metastasis of Oral Carcinoma Tongue: A Rare Case

**DOI:** 10.7759/cureus.64827

**Published:** 2024-07-18

**Authors:** Padmavathy U, Ambedkar Yadala, Bhawana Ashok Badhe

**Affiliations:** 1 Department of Radiation Oncology, Jawaharlal Institute of Postgraduate Medical Education & Research, Puducherry, IND; 2 Department of Pathology, Jawaharlal Institute of Postgraduate Medical Education & Research, Puducherry, IND

**Keywords:** radiation therapy, chemotherapy, neck, cutaneous metastasis, carcinoma tongue

## Abstract

Cutaneous metastasis from head and neck cancers is rare, typically presenting as single or multiple nodules. This report presents a truly unique and intriguing case of squamous carcinoma of the tongue, in which the patient developed numerous metastatic nodules in the face and neck, a phenomenon rarely seen in clinical practice. The patient, a known case of carcinoma tongue, was treated radically with concurrent chemoradiation. He presented with small cutaneous lesions in his neck and upper chest, which were confirmed as cutaneous malignancies. Despite receiving one cycle of palliative chemotherapy, the management of this case posed significant and complex challenges, requiring a deep understanding of the nature of the spread and metastatic pathway for choosing the appropriate management.

## Introduction

Oral cavity cancer is one of India's top three cancers, and the tongue is the most commonly involved site, accounting for 44% of all oral cancer cases [1]. Chronic alcoholism, tobacco use (including cigarettes, smokeless tobacco, and chewing betel nut), and the human papillomavirus (HPV) are the most frequent risk factors linked to oral cancer. Inadequate dental care and a bad diet can also lead to oral cancer. Lung, liver, and bones are the common sites of distant metastasis. We report a patient with carcinoma tongue who presented with cutaneous metastasis in the face, neck, and upper trunk.

## Case presentation

A 30-year-old male of Indian ethnicity, with no smoking history and occasional alcoholic consumption for six years with no comorbidities, presented with a persistent ulcer on the right side of the tongue for three months. He is a daily wage laborer of low socio-economic status with no significant family history. He was evaluated and staged as carcinoma tongue with pterygoid plate involvement and 2x1x1 cm node in the right level II cervical node, cT4bcN1cM0 AJCC staging, and HPV/p16 was not tested. The patient underwent concurrent chemoradiation treatment with a total dose of 70.4Gy over 32 fractions (EBRT) and two cycles of cisplatin chemotherapy 100 mg/m^2^ concurrently completed by April 2023. The patient tolerated chemoradiotherapy well. At the end of the treatment, the observed toxicity included grade 3 oral mucositis, grade 2 pharyngitis, and laryngitis, all managed conservatively.

**Figure 1:**
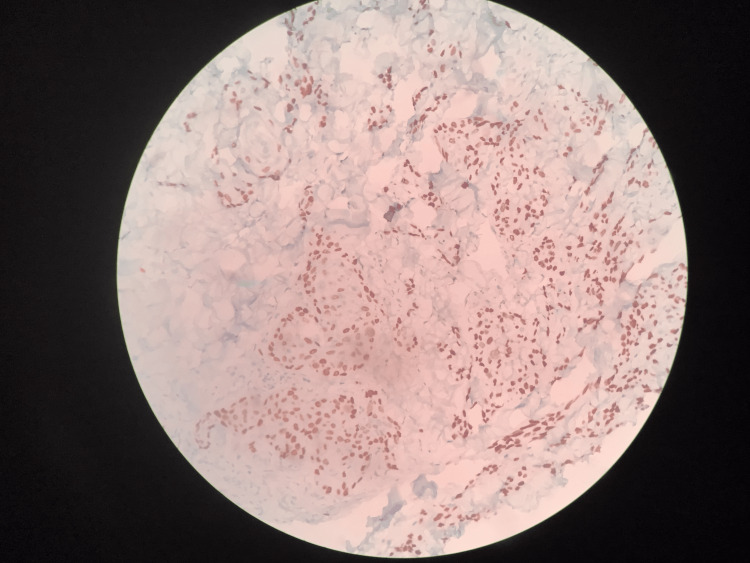
Section from the skin with deep dermis shows nests of malignant squamous cells, which are highlighted by P63 immunohistochemistry

In the first follow-up, the patient complained of small cutaneous lesions behind his right ear, which started spreading to his neck and upper chest. These lesions were initially small and painless but gradually increased in size and became tender. On local examination, multiple papules of varying sizes were slightly tender in the right post-auricular region. A few papules had ulcerated in the neck and upper chest region. Induration was present on the right lateral border of the tongue. A biopsy taken in August 2023 revealed metastatic squamous cell carcinoma in this skin lesion (Figure 1).

**Figure 2:**
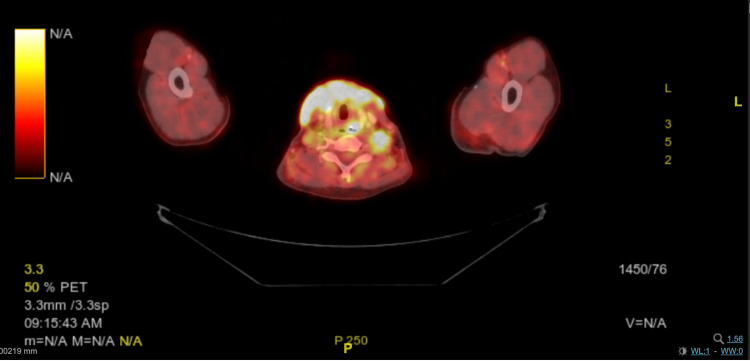
Fused image of FDG-18 PET CT showing diffuse uptake in the cutaneous region of the neck FDG-18 - Fluoro-deoxy-glucose-18; PET CT - Positron Emission Tomography-Computed Tomography

He presented with breathing difficulty (stridor), and the endoscopic exam revealed bilateral arytenoid, aryepiglottic fold, and false cord edema with critical glottic chink most likely due to radiation, not able to rule out malignancy, leading to a tracheostomy on August 2023. The PET CT scan results showed no uptake in the tongue. There was increased FDG uptake in the right lateral aspect of the neck, with a maximum standardized uptake value (SUV max) of 1.17. There was also diffuse and heterogeneous increased uptake over the skin and tissue in the front and side of the neck, with hypo-dense areas, which may be due to post-radiotherapy inflammation or possibly malignancy (Figure 2), and the tracheostomy site showed reactive changes with edema.

**Figure 3:**
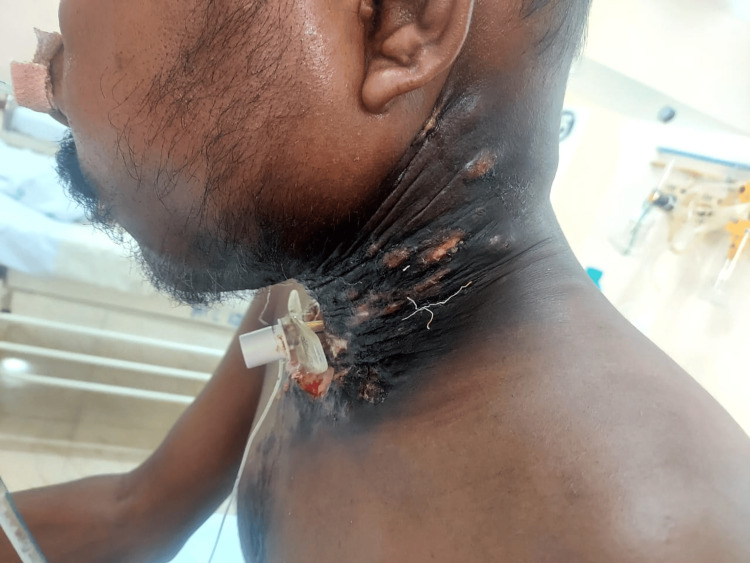
Multiple hard nodules over the lateral aspect of the neck

Subsequently, the patient developed severe facial edema on the right side of his face, and it progressed towards the left side, and his neck became woody fibrosis, as presented in Figure 3. We planned palliative chemotherapy for the patient using three weekly paclitaxel and carboplatin. Immunotherapy was not considered due to cost constraints. Despite the aggressive management, the patient's condition deteriorated rapidly. He passed away two weeks after receiving the first cycle of three weekly paclitaxel carboplatin chemotherapy.

## Discussion

The most common site for metastasis in head and neck cancer is the lung. "Cutaneous metastasis" describes how cancer cells can move from their original location to the skin. Cutaneous metastasis is highly associated with breast cancer, lung, colorectal, and ovarian cancer, and the most common age of presentation is 60 years [2,3]. They fall into three categories: loco-regional, in transit, and distant metastasis. It is of utmost challenge to confirm the cutaneous metastasis histopathologically. Most cutaneous metastases are limited to the dermis and subcutaneous adipose tissue. They can develop as linear clusters that split collagen bundles, known as "Indian filling," or as nodules or stars that grow inside or around dilated lymphatic or blood arteries. The exact mechanism was never clearly understood.

Cutaneous metastasis most commonly involves a hematogenous route, as it detaches from the primary tumor, invades and survives in circulation, extravasates, and finally gets implanted in the secondary site (i.e., skin in this case). Rastogi et al. reported multiple skin lesions in a treated case of carcinoma tongue after a disease interval of one and a half years [4]. Verma et al. reported cutaneous metastasis after four years in a treated carcinoma tongue [5]. Few reports exist for other sites, such as the larynx and hypopharynx, where the patient has developed cutaneous metastasis. Cutaneous lesions near the primary site may be due to sharing common lymphatic drainage with the primary tumor. One hypothesis is that new aberrant lymphatics can open up after irradiation, resulting in cutaneous metastasis, as seen in our case. Cutaneous metastasis usually indicates a poor prognosis [6]. Berger et al. reported a survival of about three months after skin metastasis [7]. The treatment is predominantly palliative. Our patient survived for about three months from the month of skin metastasis presentation.

## Conclusions

The incidence of carcinoma of the tongue as a type of cancer in India is relatively high, and an uncommon yet significant complication that may occur is cutaneous metastasis. This highlights the pressing need for further investigation into the mechanisms and risk factors associated with cutaneous metastasis, as well as the development of more effective treatment approaches. In such instances, the prognosis is generally unfavorable, emphasizing the critical importance of early detection and proactive intervention. This is a collective responsibility within the medical community.
